# Histone demethylase JMJD3 is required for osteoblast differentiation in mice

**DOI:** 10.1038/srep13418

**Published:** 2015-08-25

**Authors:** Feng Zhang, Longyong Xu, Longxia Xu, Qing Xu, Gerard Karsenty, Charlie Degui Chen

**Affiliations:** 1 Department of Pathology, State Key Laboratory of Cancer Biology, Xijing Hospital, Fourth Military Medical University, Changle West Road, No. 169, Xi’an, 710032, China; 2 State Key Laboratory of Molecular Biology, Shanghai Key laboratory of Molecular Andrology, Institute of Biochemistry and Cell Biology, Shanghai Institutes for Biological Sciences, Chinese Academy of Sciences, 320 Yueyang Road, Shanghai, 200031, China; 3 Department of Genetics and Development, College of Physicians and Surgeons, Columbia University, New York, NY 10032, USA

## Abstract

JMJD3 (KDM6B) is an H3K27me3 demethylases and emerges as an important player in developmental processes. Although some evidence indicated the involvement of JMJD3 in osteoblast differentiation *in vitro*, its role as a whole in osteoblast differentiation and bone formation *in vivo* remains unknown. Here we showed that homozygous deletion of *Jmjd3* resulted in severe delay of osteoblast differentiation and bone ossification in mice. By biochemical and genetical methods, we demonstrated that JMJD3 mediated RUNX2 transcriptional activity and cooperated with RUNX2 to promote osteoblast differentiation and bone formation *in vivo*. These results strongly demonstrated that JMJD3 is required for osteoblast differentiation and bone formation in mice.

During embryo development, bones form through two distinct processes: intramembranous and endochondral ossification^1^. Some cranial bones and the lateral portion of the clavicles are formed by intramembranous ossification, during which mesenchymal progenitor cells directly differentiate into bone-forming osteoblasts. In contrast, endochondral ossification involves a cartilaginous template for future bone^1^,^2^. Subsequently, this template is replaced following vascular invasion by bone cells. Meanwhile, the perichondral mesenchymal cells flanking the cartilaginous template differentiate into osteoblasts and form the periosteum or cortical bone^1^,^2^,^3^,^4^. RUNX2 is a master regulator of osteoblast differentiation for both intramembranous and endochondral ossification^1^,^2^,^3^,^4^. *RUNX2* mutation in humans led to cleidocranial dysplasia, which is characteristic of hypoplastic clavicles and delayed closure of the fontanelles^5^,^6^. *Runx2* null mice display a complete loss of both intramembranous and endochondral ossification with a blockage of osteoblast differentiation^6^,^7^.

JMJD3 is a trimethylation of histone 3 at lysine 27 (H3K27me3) specific demethylase and counteracts Polycomb-mediated transcriptional repression^8^,^9^,^10^,^11^,^12^,^13^. *Jmjd3* knockout mice showed that JMJD3 was crucial for macrophage differentiation, lung development and neurogenesis *in vivo*^14^,^15^,^16^. Recently, we observed that JMJD3 promoted chondrocyte proliferation and hypertrophy during endochondral bone formation in mice^17^. Previously, some groups showed that JMJD3 was required for osteoblast differentiation *in vitro*^18^,^19^. However, the role of JMJD3 in osteoblast differentiation and bone formation *in vivo* remains unknown. Here, we reported that JMJD3 promoted osteoblast differentiation and bone ossification *in vivo*. Moreover, we demonstrated that JMJD3 biochemically and genetically cooperates with RUNX2 to regulate osteoblast differentiation and bone ossification in mice.

## Results

### JMJD3 is required for intramembranous ossification *in vivo*

**Figure 1:**
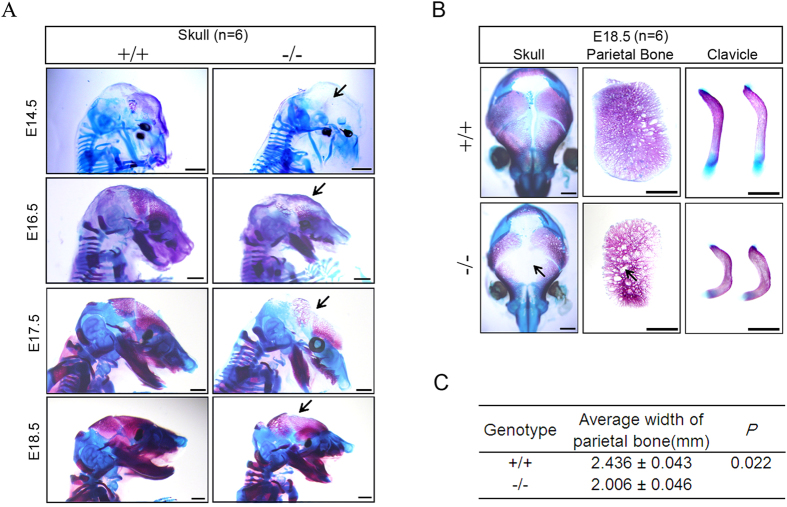
JMJD3 is required for intramembranous ossification *in vivo*. (**A**) The skeletons of E14.5, E16.5, E17.5 and E18.5 wild type and *Jmjd3*^−/−^ embryos stained with Alizarin S red (bone mineralization) and Alcian blue (cartilage). (**B**) Skull, parietal bones and clavicles of E18.5 littermates were stained by Alizarin S red and Alcian blue. (**C**) Quantitative width of the parietal bones in E18.5 WT and *Jmjd3*^−/−^ embryos. Black arrows: hypoplastic skull and parietal bones of *Jmjd3*^−/−^ embryos; n = 6. Scale bar: 1 mm.

Previously, we observed that JMJD3 is critical for chondrocyte proliferation and hypertrophy during endochondral formation^17^. To examine whether JMJD3 is also required for intramembranous ossification *in vivo*, we examined the membranous bones of E14.5–E18.5 littermates by alcian blue and alizarin S red staining. No obvious skeletal difference between WT and *Jmjd3*^+/−^ mice was detected (data not shown). However, unlike wild-type embryos, which exhibited well calcified skeletons, homozygous mutant embryos showed continually less calcification in parietal bone from E14.5 to E18.5 (Fig. 1A). Detailed investigation revealed that *Jmjd3*^−/−^ mice exhibited open fontanelles, less mineralized cranial bones and hypoplastic clavicles compared to WT littermates at E18.5 (Fig. 1B,C). These features suggested that JMJD3 is required for intramembraneous ossification *in vivo*.

### JMJD3 is required for osteoblast differentiation during both intramembraneous and endochondral bone formation

**Figure 2:**
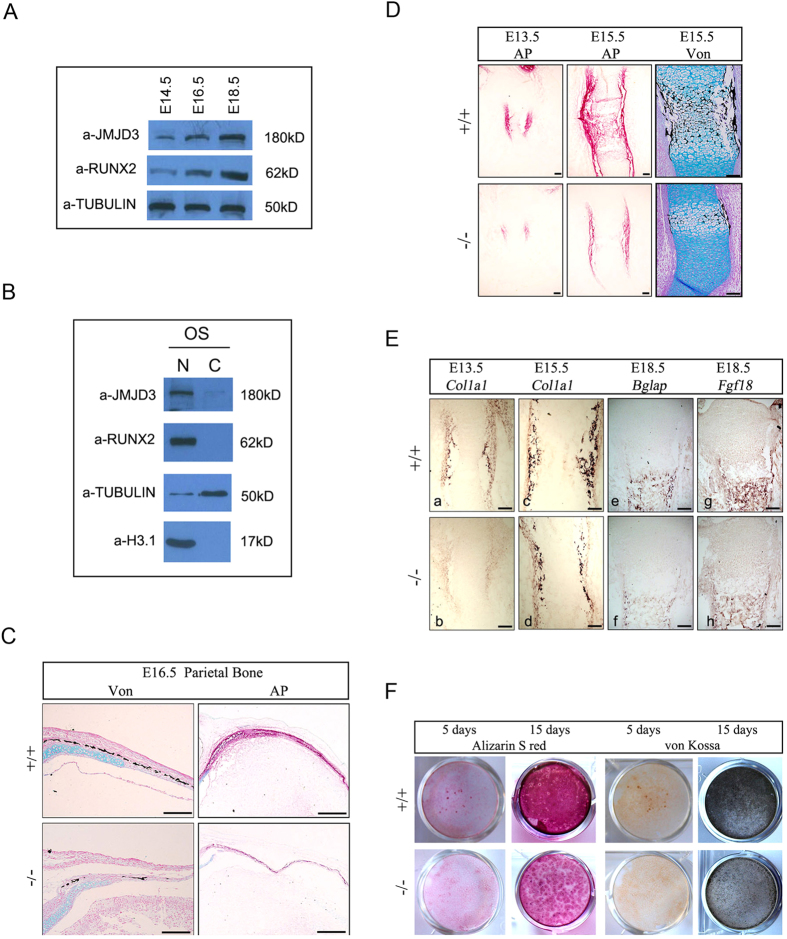
JMJD3 loss results in defects of osteoblast differentiation during both intramembraneous and endochondral bone formation. (**A**) The expression of JMJD3 and RUNX2 in primary osteoblasts from E14.5, E16.5 and E18.5 parietal bones was examined by western blot assays. (**B**) Western blot of JMJD3 in cytoplasmic (**C**) and nuclear (N) fractions of primary osteoblast (OS) from E18.5 parietal bone. RUNX2 is a positive control. (**C**) Representative von Kossa (Von) and alkaline phosphatase (AP) staining of the parietal bone sections of E16.5 WT and *Jmjd3*^−/−^ littermates. n = 3. Scale bar: 200 μm. (**D**) Von Kossa and alkaline phosphatase staining of humerus sections of WT and *Jmjd3*^−/−^ littermates at E13.5 and E15.5. n = 3. Scale bar: 200 μm. (**E**) Representative *in situ* hybridization with *Col1a1, Bglap* and *Fgf18* probes at humerus longitudinal sections of WT and *Jmjd3*^−/−^ embryos. n = 3. Scale bar: 200 μm. (**F**) *In vitro* maturation assay of primary osteoblasts isolated from parietal bones of E14.5 embryos. The osteoblasts were seeded at 1 × 10^5^ cells/cm^2^ in 6-well plates, grown to confluence and, subsequently, cultured in osteogenic media until harvest on the indicated days. Cells were then stained for Alizarin S red or von Kossa staining.

Since osteoblast maturation is essential for intramembranous ossification and endochondral bone formation, we next examined whether JMJD3 is required for osteoblast differentiation *in vivo*. Firstly, we examined the expressed level of JMJD3 in osteoblasts of E14.5, E16.5 and E18.5 parietal bones by western blot assays. The results showed that JMJD3 expression increased from E14.5 to E18.5 (Fig. 2A) and was primarily in the nuclei of osteoblasts (Fig. 2B). Subsequently, we performed histological investigations by von Kossa (Von) and alkaline phosphatase (AP) staining and observed much less mineralization and lower intensity of AP staining in the sections of parietal bones of *Jmjd3*^−/−^ embryos compared to WT littermates (Fig. 2C). Furthermore, we also observed much less mineralization and lower intensity of AP staining in *Jmjd3*^−/−^ long bones, such as in humerus (Fig. 2D). These results suggested JMJD3 is required for osteoblast maturation during both intramembraneous and endochondral bone formation.

To further characterize the defects of osteoblast differentiation *in vivo*, we performed *in situ* hybridization with RNA probes of molecular marker for osteoblast differentiation at different stages. COL1A1, the most abundant protein in the bone matrix, is an early marker of osteoblast differentiation. In E13.5 and E15.5 embryos, *in situ* hybridization indicated that *Col1a1* mRNA level was much less in the perichondrium of *Jmjd3*^−/−^ than that of WT humerui (Fig. 2Ea–d). At E18.5, the level of osteocalcin (*Bglap*), a late marker of osteoblast differentiation, markedly decreased in perichondral and periosteal osteoblasts of *Jmjd3*^−/−^ embryos compared to WT littermates (Fig. 2Ee,f). These results revealed that defects of osteoblast differentiation occurred at both early and late developmental stages of *Jmjd3*^−/−^ embryos. Consistently, the mRNA level of *Fgf18*, a factor crucial for both early and late osteoblast differentiation, also obviously reduced in mutants compared with WT embryos (Fig. 2Eg,h).

In addition, we examined the differentiation potential of primary cultured preosteoblasts from cranial bones of E14.5 WT and *Jmjd3*^−/−^ embryos in osteogenic medium. Alizarin S red and von Kossa staining revealed obvious delay of osteoblast differentiation in *Jmjd3*^−/−^ vs. WT osteoblasts (Fig. 2F). Similar results were observed in perichondral and periosteal osteoblasts derived from four limbs of E18.5 WT and *Jmjd3*^−/−^ embryos (data not shown). Collectively, these results strongly demonstrated that JMJD3 is required for osteoblast differentiation.

### JMJD3 regulates RUNX2 transcriptional activity during osteoblast differentiation

**Figure 3:**
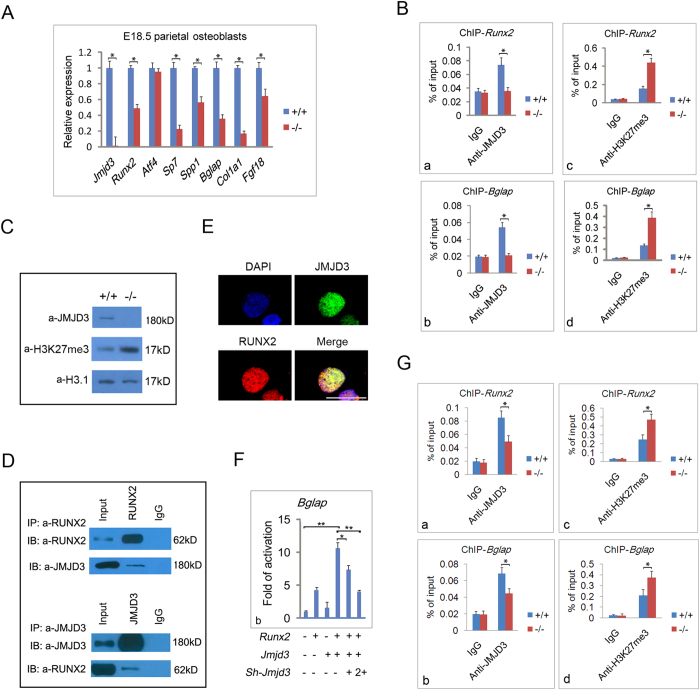
JMJD3 regulates RUNX2 transcriptional activity during osteoblast differentiation. (**A**) RT-qPCR to determine the expression levels of *Jmjd3, Runx2, Atf4, Sp7, Spp1, Bglap, Col1a1* and *Fgf18* relative to GAPDH in parietal osteoblasts of E18.5 WT and *Jmjd3*^−/−^ embryos. *p < 0.05, Error bar represents the SE of three independent experiments. (**B**) ChIP-qPCRs assays with antibodies specific for JMJD3 (a,b) or H3K27me3 (c,d) at the promoters of *Runx2* (a,c) or *Bglap* (b,d) genes in WT and *Jmjd3*^−/−^ primary parietal osteoblasts. Signals were shown as a percentage of the input. *p < 0.05, Error bar represents the SE of three independent experiments. (**C**) The total level of H3K27me3 in primary osteoblast of E18.5 WT and *Jmjd3*^−/−^ parietal bones was examined by western blot assays. (**D**) Endogenous interaction of JMJD3 and RUNX2 was examined by Co-IP assays in the primary osteoblasts from E18.5 parietal bone. IP was performed as indicated. (**E**) Immunofluorescence under confocal microscope with anti-JMJD3 (green) and anti-RUNX2 (red) antibodies on the slides of primary osteoblasts of E18.5 embryos. Scale bar: 20 μm. (**F**) Luciferase reporter assays in HEK293 cells, which were transfected with the pGL3-basic reporter containing 2 kb *Bglap* promoter with or without expression plasmids encoding *Runx2* (100 ng) , *Jmjd3* (100 ng) or sh-*Jmjd3* in increasing amounts (50, 100 ng) as indicated. The basal luciferase activity for each reporter was calculated as 1 in y axis. *p < 0.05, **p < 0.01, Error bar represents the SE of three independent experiments. (**G**) ChIP-qPCRs assays with antibodies specific for JMJD3 (a,b) or H3K27me3 (c,d) at the promoters of *Runx2* (a,c) or *Bglap* (b,d) genes in WT and *Runx2*^−/−^ primary parietal osteoblasts. Signals were shown as a percentage of the input. *p < 0.05, Error bar represents the SE of three independent experiments.

To explore the molecular mechanism of JMJD3 in osteoblast differentiation, we first analyzed expression of osteoblast-specific genes in WT and *Jmjd3*^−/−^ embryos. RT-qPCR showed that the mRNA levels of *Runx2, Sp7, Spp1, Bglap, Col1a1* and *Fgf18* in parietal osteoblasts were reduced in *Jmjd3*^−/−^ embyros compared with WT mice (Fig. 3A). In contrast, the mRNA level of *atf4* was not affected. To test whether JMJD3 directly regulates these genes transcription, we examined JMJD3 level at the promoters of *Runx2 and Bglap* by chromatin immunoprecipitation (ChIP) followed by qPCR analysis in primary osteoblasts. Indeed, JMJD3 was showed to strongly recruit to the promoters of the two genes (Fig. 3Ba,b). Consistently, the H3K27me3 level at the promoters of *Runx2* and *Bglap* genes markedly increased in *Jmjd3*^−/−^ compared with WT primary osteoblasts (Fig. 3Bc,d). These results clearly demonstrated that JMJD3 directly promoted *Runx2 and Bglap* transcription by erasing H3K27me3 in osteoblasts. This was consistent with a recent report that knockdown of *Jmjd3* in MC3T3-E1 osteoblasts resulted in increased H3K27me3 level at the promoter regions of *Runx2*^18^. In addition, western blot assay showed that knockout of *Jmjd3* increased the total level of H3K27me3 in primary osteoblasts (Fig. 3C). This indicated that JMJD3 might regulate more genes transcriptions for osteoblast differentiation.

Since RUNX2 has been shown to bind and activate its own promoter via a positive feedback loop^20^, and *Sp7, Spp1, Bglap* and *Col1a1* are target genes of RUNX2^21^, we speculated that JMJD3 may act as a coactivator of RUNX2 in osteoblast differentiation. To test this possibility, we firstly test whether JMJD3 biochemically interacts with RUNX2. Co-immunoprecipitation assays showed that RUNX2 really pull down JMJD3, or vice versa, in the primary culture of osteoblasts from E18.5 mice (Fig. 3D). In addition, immunofluorescence under confocal microscope indicated that JMJD3 co-localized with RUNX2 in the nuclei of primary osteoblasts (Fig. 3E). Next, to test whether JMJD3 can functionally cooperate with RUNX2 to regulate their target genes transcription, we performed luciferase reporter assays with *Bglap* gene promoter. The results showed that JMJD3 quite obviously synergized with RUNX2 to potentiate the expression of *Bglap*. This synergistic effect was reduced by shRNA against *Jmjd3* in a dose dependent manner (Fig. 3F). These results strongly supported that JMJD3 is a coactivator of RUNX2 in osteoblast differentiation *in vivo* and suggested that RUNX2 may recruit JMJD3 to target genes during bone formation. In order to test whether RUNX2 can affect JMJD3 binding to target genes during osteoblast differentiation, we examined JMJD3 occupancies at promoters of *Runx2* and *Bglap* genes by ChIP-qPCR in WT and *Runx2*^−*/*−^ primary osteoblasts from parietal bones. The results showed that the level of JMJD3 was reduced at the promoters of *Runx2* and *Bglap* genes in *Runx2*^−*/*−^ compared with WT osteoblasts (Fig. 3Ga,b). Consistently, the levels of H3K27me3 at the promoters of *Runx2* and *Bglap* increased in *Runx2*^−/−^ versus WT primary osteoblasts (Fig. 3Gc,d). These results supported that RUNX2 can recruit JMJD3 to target genes during osteoblast differentiation.

### JMJD3 cooperates with RUNX2 to promote osteoblast differentiation *in vivo*

**Figure 4:**
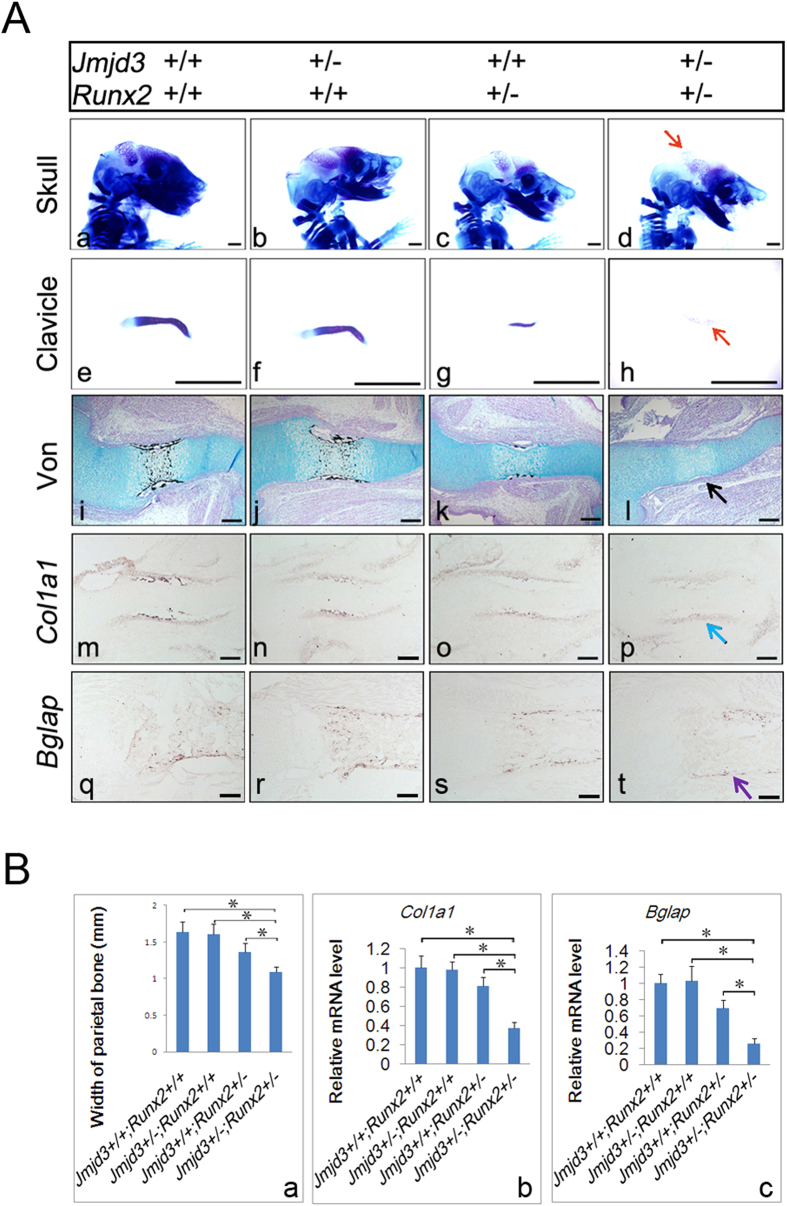
JMJD3 cooperates with RUNX2 to promote osteoblast differentiation *in vivo*. (**A**) Representative skeletal preparation, von Kossa staining and *in situ* hybridization with*Col1a1* and *Bglap* probes of *Jmjd3*^+/+^; *Runx2*^+/+^, *Jmjd3*^+/−^; *Runx2*^+/+^, *Jmjd3*^+/+^; *Runx2*^+/−^and *Jmjd*3^+/−^; *Runx2*^+/−^ mice. Red arrows: most delayed bone ossification in parietal bone and clavicles of *Jmjd*3^+/−^; *Runx2*^+/−^ mice at E15.5. Black arrow: most delayed mineralization in perichondrium of humerus of *Jmjd*3^+/−^; *Runx2*^+/−^ mice at E15.5. Blue arrow: most reduced *Col1a1* mRNA level in perichondrium of humerus of *Jmjd*3^+/−^; *Runx2*^+/−^ mice at E14.5. Purple arrow: most reduced *Bglap* mRNA level in perichondrium of humerus of *Jmjd*3^+/−^; *Runx2*^+/−^ mice at E18.5. Littermates at each group were compared. n = 3. Scale bar: 1 mm in a–h; 200 μm in i–t. (**B**) Quantification of width of parietal bones (a), *Col1a1* (b) and *Bglap* (c) mRNA level of humerus perichondrial osteoblasts of *Jmjd3*^+/+^; *Runx2*^+/+^, *Jmjd3*^+/−^; *Runx2*^+/+^, *Jmjd3*^+/+^; *Runx2*^+/−^ and *Jmjd*3^+/−^; *Runx2*^+/−^ embryos at E15.5 (a), E14.5 (b) or E18.5 (c) respectively. *p < 0.05. Error bar represents the SE of three independent experiments.

To examine whether JMJD3 cooperates with RUNX2 to promote osteoblast differentiation *in vivo*, we generated and analyzed *Jmjd3*^*+/*−^; *Runx2*^*+/*−^ embryos. Skeletal preparations examination showed that *Jmjd3*^+/−^; *Runx2*^+/−^ embryos displayed the least ossification in cranial bones (Fig. 4Ad,Ba) and clavicles (Fig. 4Ah) than WT, *Jmjd3*^+/−^, or *Runx2*^+/−^ mice at E15.5. von Kossa staining confirmed the most delay of ossification in perichondral zones of humerus of *Jmjd3*^+/−^; *Runx2*^+/−^ embryos compared with other littermates at this stage (Fig. 4Al). *In situ* hybridization and RT-qPCR for *Col1a1* and *Bglap* showed the least mRNA level at the perichondral osteoblasts of humerus of *Jmjd3*^+/−^; *Runx2*^+/−^ embryos at E14.5 or E18.5 (Fig. 4Ap,t,Bb,c). Collectively, these results clearly demonstrated that JMJD3 genetically cooperates with RUNX2 to promote osteoblast maturation during both intramembraneous and endochondral ossification, which suggested that JMJD3 is a coactivator of RUNX2 in osteoblasts.

## Discussion

Osteoblast differentiation is essential for both intramembranous and endochondral ossification. Abnormality of this process will result in various bone diseases, such as osteoporosis or osteosclerosis^22^,^23^. It has been shown previously that H3K27 methyltransferase EZH2, a core subunit of PRC2 group, suppresses mesenchymal stem cells (MSCs) from differentiating into osteoblasts^24^, while WDR5, a core component of trithorax group (TrxG), promote osteoblasts^25^. These results implicate other PRC2 and trithorax proteins may also be involved in skeleton development. As an H3k27me3 demethylases, JMJD3 counteracts the transcription repression by Polycomb group (PcG) and biochemically interacts with the core subunits of TrxG, such as WDR5, RbBP5, ASH2L and BRG1^9^,^26^. Thus, these biochemical features begged the question of the possible role of JMJD3 during osteoblast differentiation. Recently, Ye *et al.*^19^ showed that osteogenic differentiation of human bone marrow MSCs is positively regulated by JMJD3, while Yang *et al.*^18^ demonstrated that osteoblast differentiation is inhibited upon knocking-down *Jmjd3 in vitro*. In addition, both papers also showed by *in vivo* models that JMJD3 positively regulates osteogenic differentiation, either using a model injecting MSCs in which JMJD3 was silenced into mice^19^, or by finding impaired local bone formation in adult mice upon local application of siRNA against *Jmjd3* in the calvaria of living mice^18^. However, the detail roles of JMJD3 for osteoblast differentiation during both intramembranous and endochondral ossification as a whole *in vivo* is not clear. Here, using *Jmjd3* knockout mice, we demonstrated that JMJD3 is required for osteoblast differentiation during both intramembranous and endochondral ossification. This conclusion was supported by the following reasons: (1) JMJD3 was expressed in osteoblasts, (2) *Jmjd3* mutant mice displayed severe retardation of osteoblast differentiation by skeletal preparation and histological analysis, and (3) JMJD3 genetically and biochemically cooperated with RUNX2 to promote osteoblast differentiation and bone ossification *in vivo*.

The existence of a fairly typical cleidocranial dysplasia phenotype in *Jmdj3*^−*/*−^ embryos suggested that JMJD3 genetically associates with RUNX2 *in vivo*. Indeed, we detected that JMJD3 directly promotes *Runx2* transcription in osteoblasts by CHIP-qPCR assay. This was consistent with a recent report that knockdown of *Jmjd3* in MC3T3-E1 osteoblasts resulted in increased H3K27me3 level at the promoter regions of *Runx2*^18^. The result was also consistent with previous report that EZH2 directly bound to the promoter of *Runx2* to inhibit differentiation of MSCs to osteoblasts^24^. Moreover, since both *Runx2* is its own target genes^21^, we speculated that JMJD3 may act as a coactivator of RUNX2. This was verified through Co-IP and luciferase reporter assays by showing that JMJD3 favors RUNX2 transcriptional activity. More importantly, we demonstrated that JMJD3 genetically cooperated with RUNX2 to promote osteoblast differentiation and bone ossification *in vivo*. Therefore, our results not only confirmed the role of JMJD3 in osteoblast differentiation *in vivo* but also expanded the molecular mechanism of JMJD3 in bone formation. In addition, these results implicated that fine tuned counteraction of TrxG and PcG in gene transcription may play critical roles in modulating osteoblast maturation and bone development. Further studies need to explore the physiological or pathological roles of JMJD3 and other subunits of TrxG or PcG in bone biology.

## Methods

### Morphological and histology analysis

*Jmjd3*^−/−^ and *Runx2*^+/−^ mice was described previously^17^. Whole-mount staining of skeletal preparation, histological analyses, alkaline phosphatase staining and *in situ* hybridization investigations were performed as described previously^17^. Briefly, the embryos were skinned, eviscerated, and fixed in 95% ethanol. After 4 days fixation in 95% ethanol, embryos were stained in Alcian blue solution overnight. After washing with 70% ethanol, the embryos were stained by Alizarin S red solution overnight and transferred into 1% KOH for 1week. Lastly, embryos were transferred into 1% KOH/20% glycerol for 2 days and stored in 50% ethanol/50% glycerol. For histological analyses, embryos fixed in 10% formalin, processed, and embedded in paraffin or OCT (Tissue-Tek; Thermo Fisher Scientific). Serial sections were taken at 4 μm thickness. To detect mineral deposition, Von Kossa staining was performed and counterstained by alcian blue and nuclear fast red. Alkaline phosphatase staining was performed on 7 μm cryostat sections using alkaline phosphatase kit (Millpore) following the manufacturer’s instructions. For *in situ* hybridization, digoxigenin-11-UTP-labed single-stranded RNA probes were prepared with a DIG RNA labeling kit (Roche) according to the manufacturer’s instructions. *Fgf18* probe was from David Onnitz (Washington University School of Medicine) and *Col1a1* and *Bglap* probes sequences were described before^27^,^28^. All animal procedures were conducted in accordance with the Guidelines for the Care and Use of Laboratory Animals and were approved by the Institutional Animal Care and Use Committee at Shanghai Institutes for Biological Sciences.

### Primary osteoblasts culture and *in vitro* assays

Primary osteoblasts were obtained from the parietal bones or four limbs long bones of E14.5, E16.5 and E18.5 embryos by sequential collagenase A digestion at 37 °C as described^29^. Briefly, parietal bones or four limbs long bones were removed from the embryos under aseptic conditions and incubated at 37 °C in DMEM medium containing trypsin (0.5 mg/mL) for 10 min. Trypsin digests were discarded and replaced with DMEM containing 1 mg/mL of collagenase A for 20 min. The collagenase A digests were discarded and replaced with fresh enzyme dilution. The cells released at between 20 and 40 min were collected and cultured in DMEM medium (supplemented with 10% fetal calf serum). To characterize the nature of the osteoblasts, the expression of RUNX2 was examined by western blot assays. For *in vitro* maturation assays, parietal osteoblasts from E14.5 embryos at confluence were cultured in differentiation medium (a-MEM containing 10% FCS, 50 μg/mL ascorbic acid, and 5 mmol/L β-glycerolphosphate for 5 or 15 days. Cells were then stained for the presence of Alizarin S red and von Kossa staining. To characterize the nuclear localization of JMJD3 and RUNX2, double-labled immunofluorescences with anti-JMJD3 (Rabbit Polyclonal, Abcam, ab85392) and anti-RUNX2 (mouse monoclonal, Abcam, ab76956) were performed on the slides of primary osteoblasts from E18.5 embryos according to standard protocols.

### RNA isolation, reverse transcription, and real-time PCR

Parietal or perichondrial osteoblasts from E18.5 embryos were collected and lysed in TRIzol (Invitrogen) for RNA isolation following standard protocol. Reverse transcription and real-time PCR was performed as described^17^. The primer pairs used were as follows: *Jmjd3*, 5′- CAACTCCATCTGGCTGTTACTG-3′ (forward) and 5′-CCTTCTGCAACCAA- TTCCAG-3′ (reverse); *Runx2*, 5′-TGACATCCCC ATCCATCC AC-3′ (forward) and 5′-AGAAGTCAGAGGTGGCAGTG-3′ (reverse); *Col1a1*, 5′-TCCCAGAACATCACCTA- TCAC-3′ (forward) and 5′-CTGTTGCCTTCGCCTCTGAG-3′ (reverse); *Sp7*, 5′-GTCTTA- GCCA AACTCCTCTC-3′ (forward) and 5′-TCGGGAAAACGGCAAATAGG-3′ (reverse); *Spp1*, 5′-GAATGCTGTGTCCTCTGAAG-3′ (forward) and 5′-AATCCTCGCTCTCT- GCATGG-3′ (reverse); *Bgalp*, 5′-GGACCATCTTTCTGCTCACTC-3′ (forward) and 5′-CCGCTGGGCTTGGCATCTG-3′ (reverse); *Atf4,* 5′-CAAACCTTATGACCCACCTG-3′ (forward) and 5′-ACCTAGTGGCTGCTGTCTTG-3′ (reverse); *Fgf18*, 5′-GCAAGGGTC- CCAAGACCCGC-3′ (forward) and 5′-GATCCGCCGGGATCGCTTGG-3′ (reverse); *Gapdh,* 5′- CATCACAGCAACACAGAAGACC-3′ (forward) and 5′- ACCAGTAAGCTT- GCCATTGAG-3′ (reverse).

### Co-immunoprecipitation and luciferase reporter assays

To detect the interaction between endogenous JMJD3 and RUNX2, primary parietal osteoblasts from E18.5 embryos were grown to confluence on 10 × 15 cm dishs in Dulbecco’s modified Eagle’s medium (DMEM) with β-glycerophosphate (5 mmol/L) and ascorbic acid (50 μg/ml). The cells were collected and lysed in buffer as described previously^17^. Protein lysate (5 mg) was incubated for 2 hours with 3 μg anti-JMJD3 (Abcam, ab85392) or anti-RUNX2 (Cell Signaling, #8486) antibodies followed by incubation overnight with 40 μl protein A/G Plus agarose (Santa Cruz Biotechnology, Inc.). Immunoprecipitated proteins were detected by Western blotting with the anti-JMJD3 and anti-RUNX2 antibodies.

Luciferase Reporter Assays in HEK293T was described previously^17^. Briefly, HEK293T cells were cultured in DMEM media supplemented with 10% fetal bovine serum. For transient transfection assays, HEK293T cells were plated in 48-well plate at 5×10^3^/well overnight and were transfected with 100 ng pGL3-basic reporter containing *Bglap* promoter with or without expression plasmids encoding *Runx2* (100 ng), *Jmjd3* (100 ng) or *Jmjd3*-shRNA (sh-*Jmjd3*) in increasing amounts (50, 100 ng) and 10 ng *pRL-Renilla* (Promega) mixed with 1 μl Lipofectamine 2000 (Invitrogen). Cells were harvested 48 hours after transfection. For luciferase analysis, the cells were lysed according to the manufacturer’s instructions for the Dual-Luciferase Reporter assay (Promega).

### Chromatin immunoprecipitation (ChIP)

For ChIP assays, primary osteoblasts were cross linked with formaldehyde and sonicated into small chromatin fragments. Protein-DNA complexes were precipitated with rabbit anti-JMJD3 (Abcam, ab85392) or anti-H3K27me3 (Millipore, #07-449) antibodies. The purified DNA was quantified by real-time PCR with Maxima SYBR Green qPCR Master Mix. 2^△△Ct^ method was used for relative quantification. The primer pairs around of transcriptional start site used were as follows: *Runx2,* 5′-TAACGCCAGTCGGAGCAGC-3′ (forward) and 5′-CTCCCCACTTCACCCTCAG-3′ (reverse); *Bglap*, 5′-TGAAACCAGATACCCCCGAG-3′ (forward) and 5′-GCCCTGCTTG- TGTTGGAGAC-3′.

### Statistical analyses

All quantitative data are presented as mean ± standard error (SE) with a minimum of three independent samples. Statistical significance is determined by two-tailed Student’s t-test.

## Additional Information

**How to cite this article**: Zhang, F. *et al.* Histone demethylase JMJD3 is required for osteoblast differentiation in mice. *Sci. Rep.* **5**, 13418; doi: 10.1038/srep13418 (2015).
